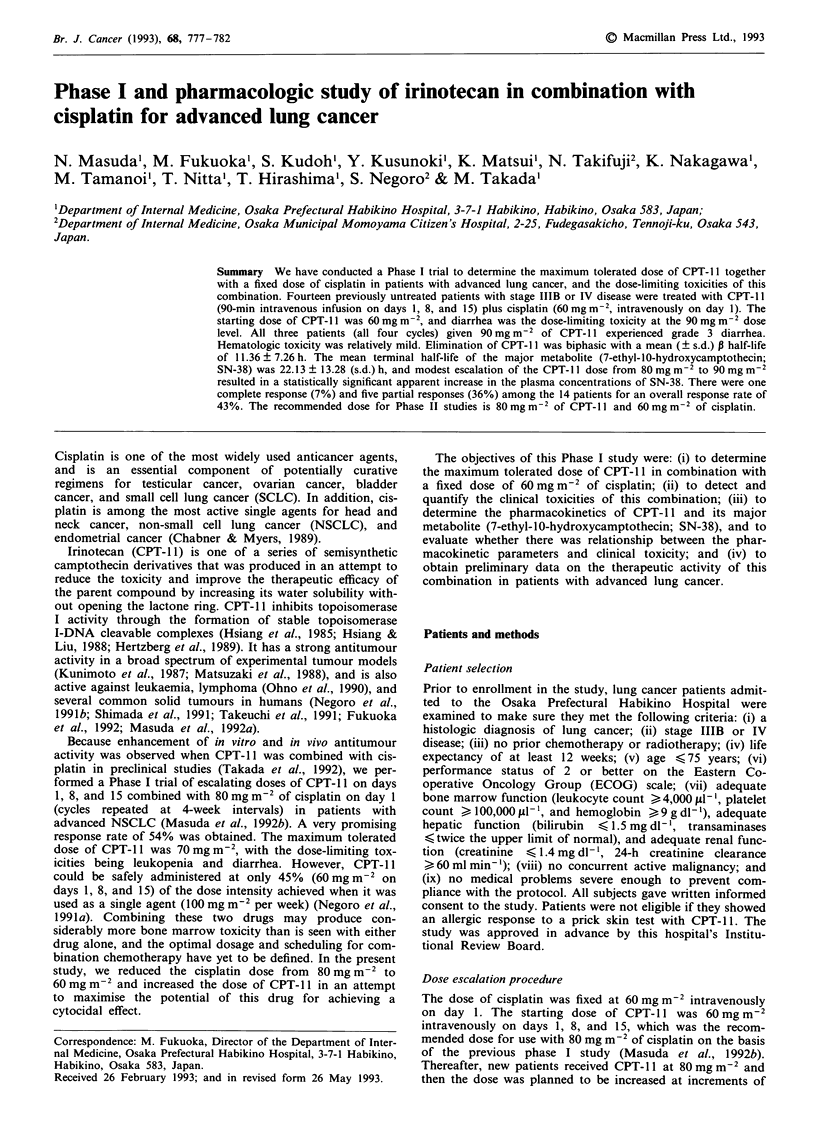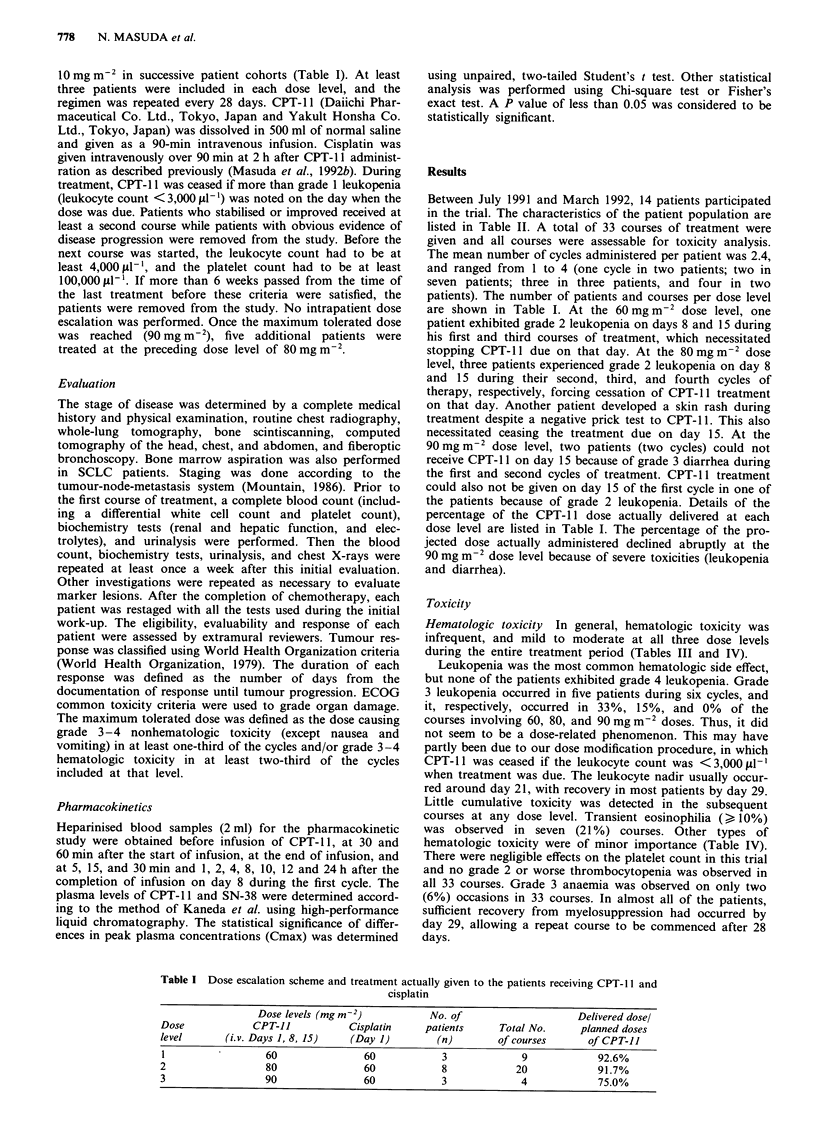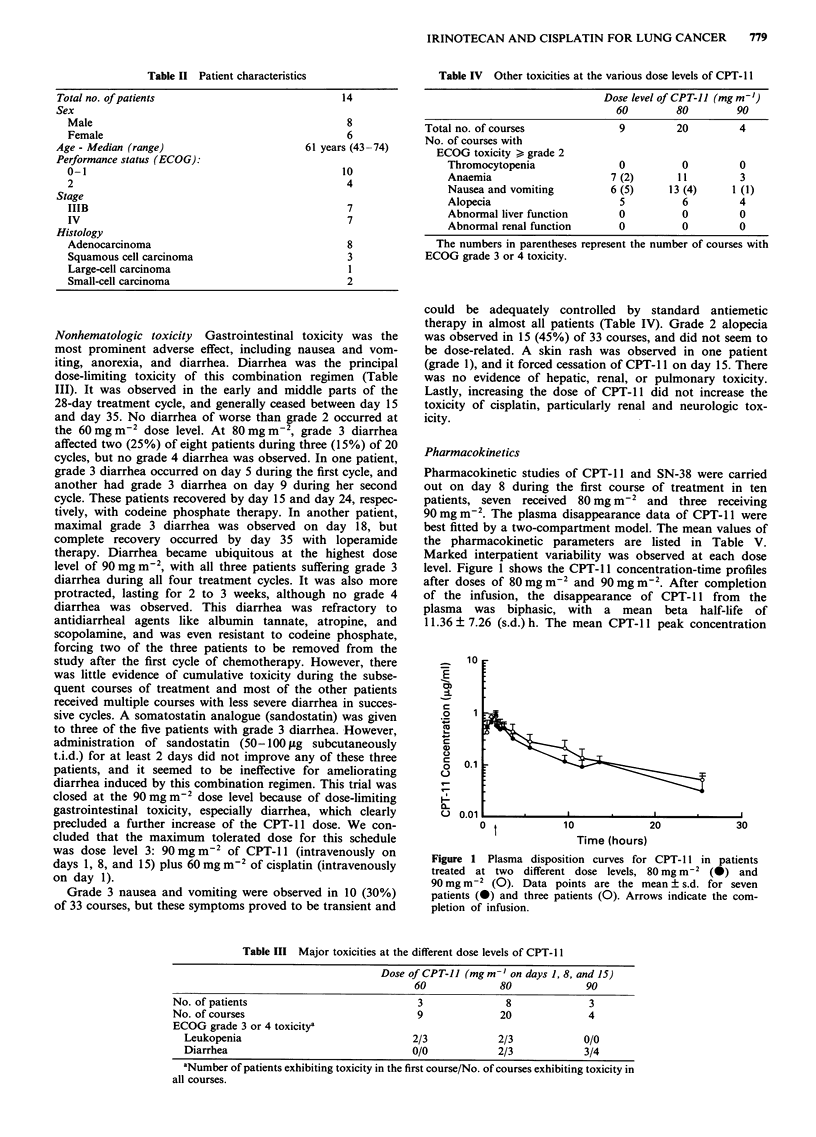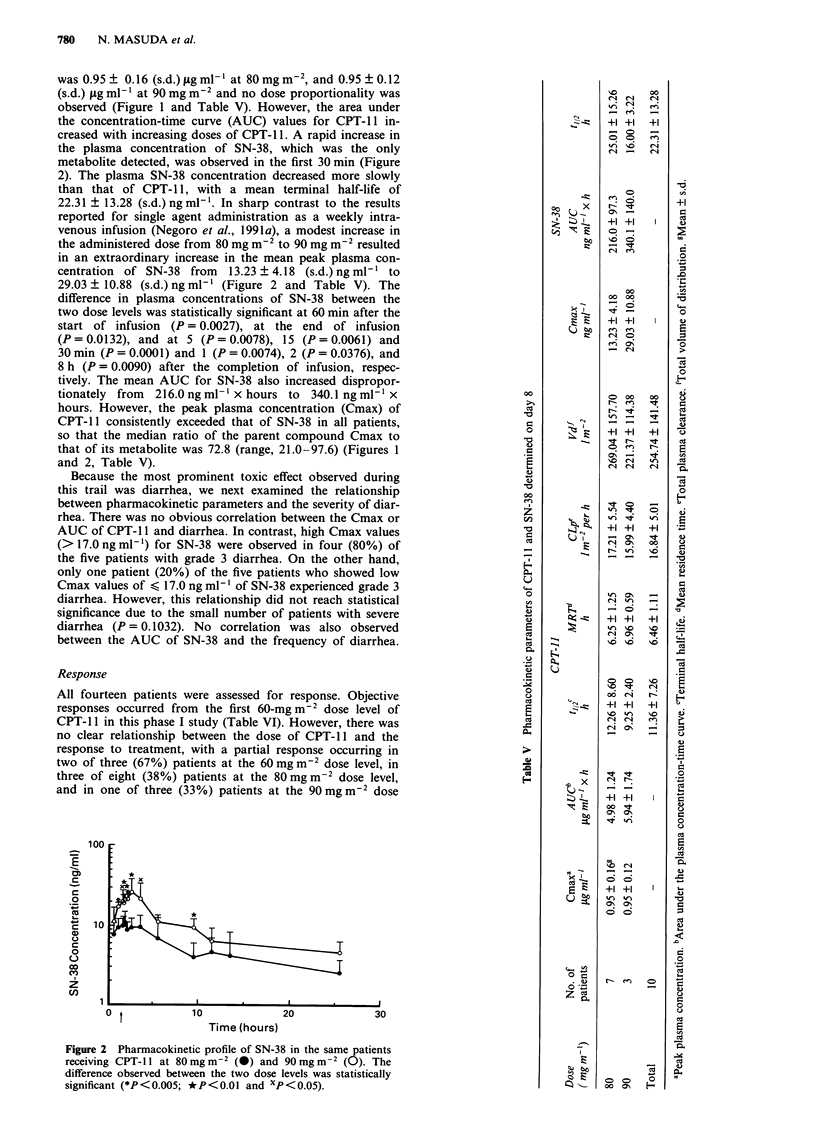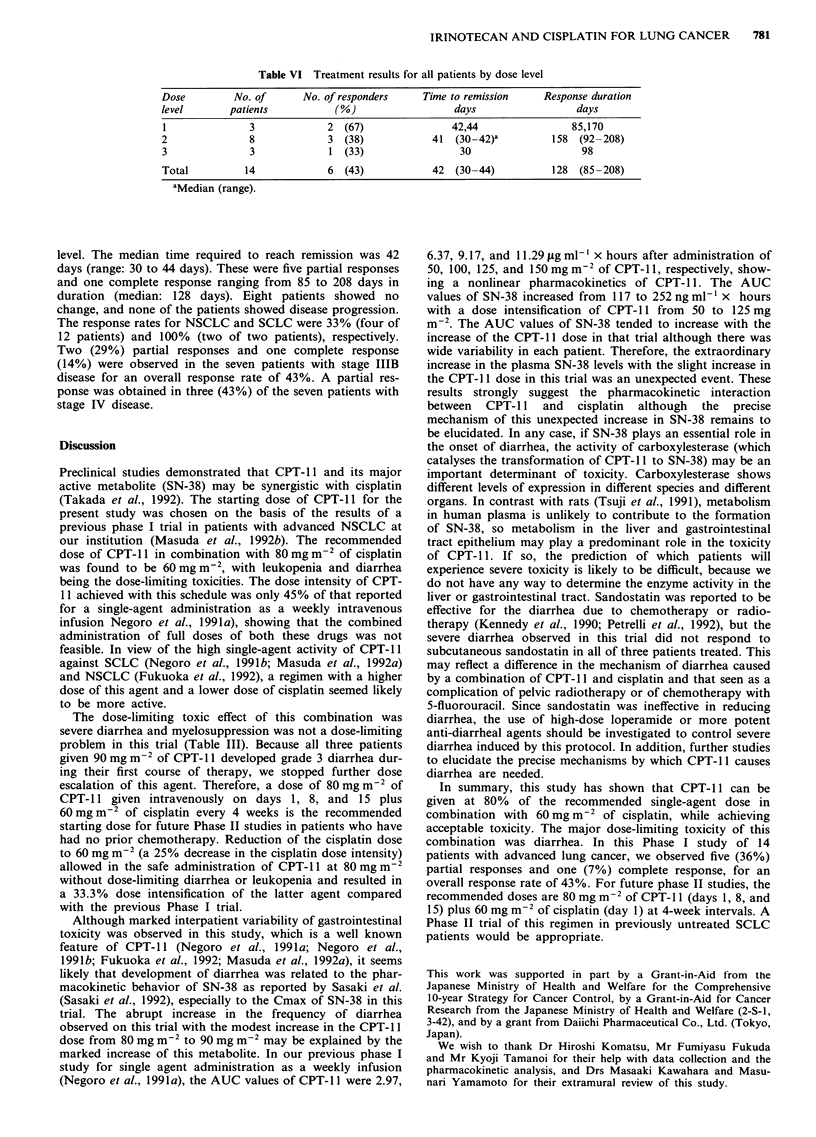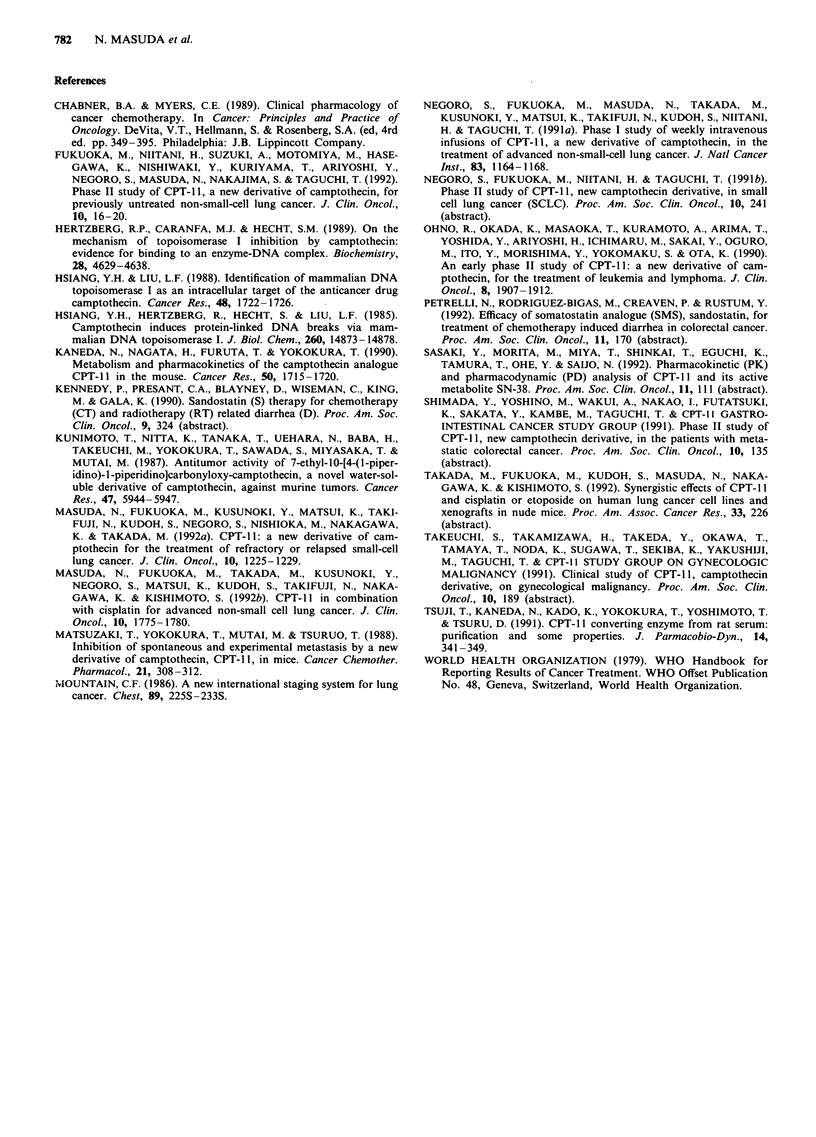# Phase I and pharmacologic study of irinotecan in combination with cisplatin for advanced lung cancer.

**DOI:** 10.1038/bjc.1993.427

**Published:** 1993-10

**Authors:** N. Masuda, M. Fukuoka, S. Kudoh, Y. Kusunoki, K. Matsui, N. Takifuji, K. Nakagawa, M. Tamanoi, T. Nitta, T. Hirashima

**Affiliations:** Department of Internal Medicine, Osaka Prefectural Habikino Hospital, Osaka, Japan.

## Abstract

We have conducted a Phase I trial to determine the maximum tolerated dose of CPT-11 together with a fixed dose of cisplatin in patients with advanced lung cancer, and the dose-limiting toxicities of this combination. Fourteen previously untreated patients with stage IIIB or IV disease were treated with CPT-11 (90-min intravenous infusion on days 1, 8, and 15) plus cisplatin (60 mg m-2, intravenously on day 1). The starting dose of CPT-11 was 60 mg m-2, and diarrhea was the dose-limiting toxicity at the 90 mg m-2 dose level. All three patients (all four cycles) given 90 mg m-2 of CPT-11 experienced grade 3 diarrhea. Hematologic toxicity was relatively mild. Elimination of CPT-11 was biphasic with a mean (+/- s.d.) beta half-life of 11.36 +/- 7.26 h. The mean terminal half-life of the major metabolite (7-ethyl-10-hydroxycamptothecin; SN-38) was 22.13 +/- 13.28 (s.d.) h, and modest escalation of the CPT-11 dose from 80 mg m-2 to 90 mg m-2 resulted in a statistically significant apparent increase in the plasma concentrations of SN-38. There were one complete response (7%) and five partial responses (36%) among the 14 patients for an overall response rate of 43%. The recommended dose for Phase II studies is 80 mg m-2 of CPT-11 and 60 mg m-2 of cisplatin.


Br. J. Cancer (1993), 68, 777-782 © Macmillan Press Ltd., 1993

### Phase I and pharmacologic study of irinotecan in combination with cisplatin for advanced lung cancer

N. Masuda[1], M. Fukuoka[1], S. Kudoh[1], Y. Kusunoki[1], K. Matsui[1], N. Takifuji[2], K. Nakagawa[1], M. Tamanoi[1], T. Nitta[1], T. Hirashima[1], S. Negoro[2] & M. Takada[1]

[1]*Department of Internal Medicine, Osaka Prefectural Habikino Hospital, 3-7-1 Habikino, Habikino, Osaka 583, Japan;*
[2]*Department of Internal Medicine, Osaka Municipal Momoyama Citizen's Hospital, 2-25, Fudegasakicho, Tennoji-ku, Osaka 543, Japan.*

**Summary** We have conducted a Phase I trial to determine the maximum tolerated dose of CPT-11 together with a fixed dose of cisplatin in patients with advanced lung cancer, and the dose-limiting toxicities of this combination. Fourteen previously untreated patients with stage IIIB or IV disease were treated with CPT-11 (90-min intravenous infusion on days 1, 8, and 15) plus cisplatin (60 mg m$^{-2}$, intravenously on day 1). The starting dose of CPT-11 was 60 mg m$^{-2}$, and diarrhea was the dose-limiting toxicity at the 90 mg m$^{-2}$ dose level. All three patients (all four cycles) given 90 mg m$^{-2}$ of CPT-11 experienced grade 3 diarrhea. Hematologic toxicity was relatively mild. Elimination of CPT-11 was biphasic with a mean (± s.d.) $\beta$ half-life of 11.36 ± 7.26 h. The mean terminal half-life of the major metabolite (7-ethyl-10-hydroxycamptothecin; SN-38) was 22.13 ± 13.28 (s.d.) h, and modest escalation of the CPT-11 dose from 80 mg m$^{-2}$ to 90 mg m$^{-2}$ resulted in a statistically significant apparent increase in the plasma concentrations of SN-38. There were one complete response (7%) and five partial responses (36%) among the 14 patients for an overall response rate of 43%. The recommended dose for Phase II studies is 80 mg m$^{-2}$ of CPT-11 and 60 mg m$^{-2}$ of cisplatin.

Cisplatin is one of the most widely used anticancer agents, and is an essential component of potentially curative regimens for testicular cancer, ovarian cancer, bladder cancer, and small cell lung cancer (SCLC). In addition, cisplatin is among the most active single agents for head and neck cancer, non-small cell lung cancer (NSCLC), and endometrial cancer (Chabner & Myers, 1989).

Irinotecan (CPT-11) is one of a series of semisynthetic camptothecin derivatives that was produced in an attempt to reduce the toxicity and improve the therapeutic efficacy of the parent compound by increasing its water solubility without opening the lactone ring. CPT-11 inhibits topoisomerase I activity through the formation of stable topoisomerase I-DNA cleavable complexes (Hsiang *et al.*, 1985; Hsiang & Liu, 1988; Hertzberg *et al.*, 1989). It has a strong antitumour activity in a broad spectrum of experimental tumour models (Kunimoto *et al.*, 1987; Matsuzaki *et al.*, 1988), and is also active against leukaemia, lymphoma (Ohno *et al.*, 1990), and several common solid tumours in humans (Negoro *et al.*, 1991*b*; Shimada *et al.*, 1991; Takeuchi *et al.*, 1991; Fukuoka *et al.*, 1992; Masuda *et al.*, 1992*a*).

Because enhancement of *in vitro* and *in vivo* antitumour activity was observed when CPT-11 was combined with cisplatin in preclinical studies (Takada *et al.*, 1992), we performed a Phase I trial of escalating doses of CPT-11 on days 1, 8, and 15 combined with 80 mg m$^{-2}$ of cisplatin on day 1 (cycles repeated at 4-week intervals) in patients with advanced NSCLC (Masuda *et al.*, 1992*b*). A very promising response rate of 54% was obtained. The maximum tolerated dose of CPT-11 was 70 mg m$^{-2}$, with the dose-limiting toxicities being leukopenia and diarrhea. However, CPT-11 could be safely administered at only 45% (60 mg m$^{-2}$ on days 1, 8, and 15) of the dose intensity achieved when it was used as a single agent (100 mg m$^{-2}$ per week) (Negoro *et al.*, 1991*a*). Combining these two drugs may produce considerably more bone marrow toxicity than is seen with either drug alone, and the optimal dosage and scheduling for combination chemotherapy have yet to be defined. In the present study, we reduced the cisplatin dose from 80 mg m$^{-2}$ to 60 mg m$^{-2}$ and increased the dose of CPT-11 in an attempt to maximise the potential of this drug for achieving a cytocidal effect.

Correspondence: M. Fukuoka, Director of the Department of Internal Medicine, Osaka Prefectural Habikino Hospital, 3-7-1 Habikino, Habikino, Osaka 583, Japan.
Received 26 February 1993; and in revised form 26 May 1993.

The objectives of this Phase I study were: (i) to determine the maximum tolerated dose of CPT-11 in combination with a fixed dose of 60 mg m$^{-2}$ of cisplatin; (ii) to detect and quantify the clinical toxicities of this combination; (iii) to determine the pharmacokinetics of CPT-11 and its major metabolite (7-ethyl-10-hydroxycamptothecin; SN-38), and to evaluate whether there was relationship between the pharmacokinetic parameters and clinical toxicity; and (iv) to obtain preliminary data on the therapeutic activity of this combination in patients with advanced lung cancer.

### Patients and methods

### *Patient selection*

Prior to enrollment in the study, lung cancer patients admitted to the Osaka Prefectural Habikino Hospital were examined to make sure they met the following criteria: (i) a histologic diagnosis of lung cancer; (ii) stage IIIB or IV disease; (iii) no prior chemotherapy or radiotherapy; (iv) life expectancy of at least 12 weeks; (v) age $\leqslant$75 years; (vi) performance status of 2 or better on the Eastern Cooperative Oncology Group (ECOG) scale; (vii) adequate bone marrow function (leukocyte count $\geqslant$4,000 $\mu$l$^{-1}$, platelet count $\geqslant$100,000 $\mu$l$^{-1}$, and hemoglobin $\geqslant$9 g dl$^{-1}$), adequate hepatic function (bilirubin $\leqslant$1.5 mg dl$^{-1}$, transaminases $\leqslant$twice the upper limit of normal), and adequate renal function (creatinine $\leqslant$1.4 mg dl$^{-1}$, 24-h creatinine clearance $\geqslant$60 ml min$^{-1}$); (viii) no concurrent active malignancy; and (ix) no medical problems severe enough to prevent compliance with the protocol. All subjects gave written informed consent to the study. Patients were not eligible if they showed an allergic response to a prick skin test with CPT-11. The study was approved in advance by this hospital's Institutional Review Board.

### *Dose escalation procedure*

The dose of cisplatin was fixed at 60 mg m$^{-2}$ intravenously on day 1. The starting dose of CPT-11 was 60 mg m$^{-2}$ intravenously on days 1, 8, and 15, which was the recommended dose for use with 80 mg m$^{-2}$ of cisplatin on the basis of the previous phase I study (Masuda *et al.*, 1992*b*). Thereafter, new patients received CPT-11 at 80 mg m$^{-2}$ and then the dose was planned to be increased at increments of

10 mg m$^{-2}$ in successive patient cohorts (Table I). At least three patients were included in each dose level, and the regimen was repeated every 28 days. CPT-11 (Daiichi Pharmaceutical Co. Ltd., Tokyo, Japan and Yakult Honsha Co. Ltd., Tokyo, Japan) was dissolved in 500 ml of normal saline and given as a 90-min intravenous infusion. Cisplatin was given intravenously over 90 min at 2 h after CPT-11 administration as described previously (Masuda *et al.*, 1992*b*). During treatment, CPT-11 was ceased if more than grade 1 leukopenia (leukocyte count $< 3,000\ \mu l^{-1}$) was noted on the day when the dose was due. Patients who stabilised or improved received at least a second course while patients with obvious evidence of disease progression were removed from the study. Before the next course was started, the leukocyte count had to be at least 4,000 $\mu l^{-1}$, and the platelet count had to be at least 100,000 $\mu l^{-1}$. If more than 6 weeks passed from the time of the last treatment before these criteria were satisfied, the patients were removed from the study. No intrapatient dose escalation was performed. Once the maximum tolerated dose was reached (90 mg m$^{-2}$), five additional patients were treated at the preceding dose level of 80 mg m$^{-2}$.

### *Evaluation*

The stage of disease was determined by a complete medical history and physical examination, routine chest radiography, whole-lung tomography, bone scintiscanning, computed tomography of the head, chest, and abdomen, and fiberoptic bronchoscopy. Bone marrow aspiration was also performed in SCLC patients. Staging was done according to the tumour-node-metastasis system (Mountain, 1986). Prior to the first course of treatment, a complete blood count (including a differential white cell count and platelet count), biochemistry tests (renal and hepatic function, and electrolytes), and urinalysis were performed. Then the blood count, biochemistry tests, urinalysis, and chest X-rays were repeated at least once a week after this initial evaluation. Other investigations were repeated as necessary to evaluate marker lesions. After the completion of chemotherapy, each patient was restaged with all the tests used during the initial work-up. The eligibility, evaluability and response of each patient were assessed by extramural reviewers. Tumour response was classified using World Health Organization criteria (World Health Organization, 1979). The duration of each response was defined as the number of days from the documentation of response until tumour progression. ECOG common toxicity criteria were used to grade organ damage. The maximum tolerated dose was defined as the dose causing grade 3–4 nonhematologic toxicity (except nausea and vomiting) in at least one-third of the cycles and/or grade 3–4 hematologic toxicity in at least two-third of the cycles included at that level.

### *Pharmacokinetics*

Heparinised blood samples (2 ml) for the pharmacokinetic study were obtained before infusion of CPT-11, at 30 and 60 min after the start of infusion, at the end of infusion, and at 5, 15, and 30 min and 1, 2, 4, 8, 10, 12 and 24 h after the completion of infusion on day 8 during the first cycle. The plasma levels of CPT-11 and SN-38 were determined according to the method of Kaneda *et al.* using high-performance liquid chromatography. The statistical significance of differences in peak plasma concentrations (Cmax) was determined using unpaired, two-tailed Student's *t* test. Other statistical analysis was performed using Chi-square test or Fisher's exact test. A $P$ value of less than 0.05 was considered to be statistically significant.

### Results

Between July 1991 and March 1992, 14 patients participated in the trial. The characteristics of the patient population are listed in Table II. A total of 33 courses of treatment were given and all courses were assessable for toxicity analysis. The mean number of cycles administered per patient was 2.4, and ranged from 1 to 4 (one cycle in two patients; two in seven patients; three in three patients, and four in two patients). The number of patients and courses per dose level are shown in Table I. At the 60 mg m$^{-2}$ dose level, one patient exhibited grade 2 leukopenia on days 8 and 15 during his first and third courses of treatment, which necessitated stopping CPT-11 due on that day. At the 80 mg m$^{-2}$ dose level, three patients experienced grade 2 leukopenia on day 8 and 15 during their second, third, and fourth cycles of therapy, respectively, forcing cessation of CPT-11 treatment on that day. Another patient developed a skin rash during treatment despite a negative prick test to CPT-11. This also necessitated ceasing the treatment due on day 15. At the 90 mg m$^{-2}$ dose level, two patients (two cycles) could not receive CPT-11 on day 15 because of grade 3 diarrhea during the first and second cycles of treatment. CPT-11 treatment could also not be given on day 15 of the first cycle in one of the patients because of grade 2 leukopenia. Details of the percentage of the CPT-11 dose actually delivered at each dose level are listed in Table I. The percentage of the projected dose actually administered declined abruptly at the 90 mg m$^{-2}$ dose level because of severe toxicities (leukopenia and diarrhea).

### *Toxicity*

*Hematologic toxicity* In general, hematologic toxicity was infrequent, and mild to moderate at all three dose levels during the entire treatment period (Tables III and IV).

Leukopenia was the most common hematologic side effect, but none of the patients exhibited grade 4 leukopenia. Grade 3 leukopenia occurred in five patients during six cycles, and it, respectively, occurred in 33%, 15%, and 0% of the courses involving 60, 80, and 90 mg m$^{-2}$ doses. Thus, it did not seem to be a dose-related phenomenon. This may have partly been due to our dose modification procedure, in which CPT-11 was ceased if the leukocyte count was $< 3,000\ \mu l^{-1}$ when treatment was due. The leukocyte nadir usually occurred around day 21, with recovery in most patients by day 29. Little cumulative toxicity was detected in the subsequent courses at any dose level. Transient eosinophilia ($\geqslant 10\%$) was observed in seven (21%) courses. Other types of hematologic toxicity were of minor importance (Table IV). There were negligible effects on the platelet count in this trial and no grade 2 or worse thrombocytopenia was observed in all 33 courses. Grade 3 anaemia was observed on only two (6%) occasions in 33 courses. In almost all of the patients, sufficient recovery from myelosuppression had occurred by day 29, allowing a repeat course to be commenced after 28 days.

**Table I** Dose escalation scheme and treatment actually given to the patients receiving CPT-11 and cisplatin

| Dose level | Dose levels (mg m$^{-2}$) CPT-11 (i.v. Days 1, 8, 15) | Cisplatin (Day 1) | No. of patients (n) | Total No. of courses | Delivered dose/planned doses of CPT-11 |
|---|---|---|---|---|---|
| 1 | 60 | 60 | 3 | 9 | 92.6% |
| 2 | 80 | 60 | 8 | 20 | 91.7% |
| 3 | 90 | 60 | 3 | 4 | 75.0% |

**Table II** Patient characteristics

| | |
|---|---|
| *Total no. of patients* | 14 |
| *Sex* | |
| Male | 8 |
| Female | 6 |
| *Age - Median (range)* | 61 years (43–74) |
| *Performance status (ECOG):* | |
| 0–1 | 10 |
| 2 | 4 |
| *Stage* | |
| IIIB | 7 |
| IV | 7 |
| *Histology* | |
| Adenocarcinoma | 8 |
| Squamous cell carcinoma | 3 |
| Large-cell carcinoma | 1 |
| Small-cell carcinoma | 2 |

*Nonhematologic toxicity* Gastrointestinal toxicity was the most prominent adverse effect, including nausea and vomiting, anorexia, and diarrhea. Diarrhea was the principal dose-limiting toxicity of this combination regimen (Table III). It was observed in the early and middle parts of the 28-day treatment cycle, and generally ceased between day 15 and day 35. No diarrhea of worse than grade 2 occurred at the 60 mg $m^{-2}$ dose level. At 80 mg $m^{-2}$, grade 3 diarrhea affected two (25%) of eight patients during three (15%) of 20 cycles, but no grade 4 diarrhea was observed. In one patient, grade 3 diarrhea occurred on day 5 during the first cycle, and another had grade 3 diarrhea on day 9 during her second cycle. These patients recovered by day 15 and day 24, respectively, with codeine phosphate therapy. In another patient, maximal grade 3 diarrhea was observed on day 18, but complete recovery occurred by day 35 with loperamide therapy. Diarrhea became ubiquitous at the highest dose level of 90 mg $m^{-2}$, with all three patients suffering grade 3 diarrhea during all four treatment cycles. It was also more protracted, lasting for 2 to 3 weeks, although no grade 4 diarrhea was observed. This diarrhea was refractory to antidiarrheal agents like albumin tannate, atropine, and scopolamine, and was even resistant to codeine phosphate, forcing two of the three patients to be removed from the study after the first cycle of chemotherapy. However, there was little evidence of cumulative toxicity during the subsequent courses of treatment and most of the other patients received multiple courses with less severe diarrhea in successive cycles. A somatostatin analogue (sandostatin) was given to three of the five patients with grade 3 diarrhea. However, administration of sandostatin (50–100 µg subcutaneously t.i.d.) for at least 2 days did not improve any of these three patients, and it seemed to be ineffective for ameliorating diarrhea induced by this combination regimen. This trial was closed at the 90 mg $m^{-2}$ dose level because of dose-limiting gastrointestinal toxicity, especially diarrhea, which clearly precluded a further increase of the CPT-11 dose. We concluded that the maximum tolerated dose for this schedule was dose level 3: 90 mg $m^{-2}$ of CPT-11 (intravenously on days 1, 8, and 15) plus 60 mg $m^{-2}$ of cisplatin (intravenously on day 1).

Grade 3 nausea and vomiting were observed in 10 (30%) of 33 courses, but these symptoms proved to be transient and could be adequately controlled by standard antiemetic therapy in almost all patients (Table IV). Grade 2 alopecia was observed in 15 (45%) of 33 courses, and did not seem to be dose-related. A skin rash was observed in one patient (grade 1), and it forced cessation of CPT-11 on day 15. There was no evidence of hepatic, renal, or pulmonary toxicity. Lastly, increasing the dose of CPT-11 did not increase the toxicity of cisplatin, particularly renal and neurologic toxicity.

**Table IV** Other toxicities at the various dose levels of CPT-11

| | *Dose level of CPT-11 (mg $m^{-1}$)* | | |
|---|---|---|---|
| | *60* | *80* | *90* |
| Total no. of courses | 9 | 20 | 4 |
| No. of courses with ECOG toxicity ⩾ grade 2 | | | |
| Thromocytopenia | 0 | 0 | 0 |
| Anaemia | 7 (2) | 11 | 3 |
| Nausea and vomiting | 6 (5) | 13 (4) | 1 (1) |
| Alopecia | 5 | 6 | 4 |
| Abnormal liver function | 0 | 0 | 0 |
| Abnormal renal function | 0 | 0 | 0 |

The numbers in parentheses represent the number of courses with ECOG grade 3 or 4 toxicity.

*Pharmacokinetics*

Pharmacokinetic studies of CPT-11 and SN-38 were carried out on day 8 during the first course of treatment in ten patients, seven received 80 mg $m^{-2}$ and three receiving 90 mg $m^{-2}$. The plasma disappearance data of CPT-11 were best fitted by a two-compartment model. The mean values of the pharmacokinetic parameters are listed in Table V. Marked interpatient variability was observed at each dose level. Figure 1 shows the CPT-11 concentration-time profiles after doses of 80 mg $m^{-2}$ and 90 mg $m^{-2}$. After completion of the infusion, the disappearance of CPT-11 from the plasma was biphasic, with a mean beta half-life of 11.36 ± 7.26 (s.d.) h. The mean CPT-11 peak concentration

**Figure 1** Plasma disposition curves for CPT-11 in patients treated at two different dose levels, 80 mg $m^{-2}$ (●) and 90 mg $m^{-2}$ (○). Data points are the mean ± s.d. for seven patients (●) and three patients (○). Arrows indicate the completion of infusion.

**Table III** Major toxicities at the different dose levels of CPT-11

| | *Dose of CPT-11 (mg $m^{-1}$ on days 1, 8, and 15)* | | |
|---|---|---|---|
| | *60* | *80* | *90* |
| No. of patients | 3 | 8 | 3 |
| No. of courses | 9 | 20 | 4 |
| ECOG grade 3 or 4 toxicity[a] | | | |
| Leukopenia | 2/3 | 2/3 | 0/0 |
| Diarrhea | 0/0 | 2/3 | 3/4 |

[a]Number of patients exhibiting toxicity in the first course/No. of courses exhibiting toxicity in all courses.

was 0.95 ± 0.16 (s.d.) μg ml$^{-1}$ at 80 mg m$^{-2}$, and 0.95 ± 0.12 (s.d.) μg ml$^{-1}$ at 90 mg m$^{-2}$ and no dose proportionality was observed (Figure 1 and Table V). However, the area under the concentration-time curve (AUC) values for CPT-11 increased with increasing doses of CPT-11. A rapid increase in the plasma concentration of SN-38, which was the only metabolite detected, was observed in the first 30 min (Figure 2). The plasma SN-38 concentration decreased more slowly than that of CPT-11, with a mean terminal half-life of 22.31 ± 13.28 (s.d.) ng ml$^{-1}$. In sharp contrast to the results reported for single agent administration as a weekly intravenous infusion (Negoro et al., 1991a), a modest increase in the administered dose from 80 mg m$^{-2}$ to 90 mg m$^{-2}$ resulted in an extraordinary increase in the mean peak plasma concentration of SN-38 from 13.23 ± 4.18 (s.d.) ng ml$^{-1}$ to 29.03 ± 10.88 (s.d.) ng ml$^{-1}$ (Figure 2 and Table V). The difference in plasma concentrations of SN-38 between the two dose levels was statistically significant at 60 min after the start of infusion ($P = 0.0027$), at the end of infusion ($P = 0.0132$), and at 5 ($P = 0.0078$), 15 ($P = 0.0061$) and 30 min ($P = 0.0001$) and 1 ($P = 0.0074$), 2 ($P = 0.0376$), and 8 h ($P = 0.0090$) after the completion of infusion, respectively. The mean AUC for SN-38 also increased disproportionately from 216.0 ng ml$^{-1}$ × hours to 340.1 ng ml$^{-1}$ × hours. However, the peak plasma concentration (Cmax) of CPT-11 consistently exceeded that of SN-38 in all patients, so that the median ratio of the parent compound Cmax to that of its metabolite was 72.8 (range, 21.0–97.6) (Figures 1 and 2, Table V).

Because the most prominent toxic effect observed during this trail was diarrhea, we next examined the relationship between pharmacokinetic parameters and the severity of diarrhea. There was no obvious correlation between the Cmax or AUC of CPT-11 and diarrhea. In contrast, high Cmax values (> 17.0 ng ml$^{-1}$) for SN-38 were observed in four (80%) of the five patients with grade 3 diarrhea. On the other hand, only one patient (20%) of the five patients who showed low Cmax values of ⩽ 17.0 ng ml$^{-1}$ of SN-38 experienced grade 3 diarrhea. However, this relationship did not reach statistical significance due to the small number of patients with severe diarrhea ($P = 0.1032$). No correlation was also observed between the AUC of SN-38 and the frequency of diarrhea.

*Response*

All fourteen patients were assessed for response. Objective responses occurred from the first 60-mg m$^{-2}$ dose level of CPT-11 in this phase I study (Table VI). However, there was no clear relationship between the dose of CPT-11 and the response to treatment, with a partial response occurring in two of three (67%) patients at the 60 mg m$^{-2}$ dose level, in three of eight (38%) patients at the 80 mg m$^{-2}$ dose level, and in one of three (33%) patients at the 90 mg m$^{-2}$ dose

**Figure 2** Pharmacokinetic profile of SN-38 in the same patients receiving CPT-11 at 80 mg m$^{-2}$ (●) and 90 mg m$^{-2}$ (○). The difference observed between the two dose levels was statistically significant (*$P < 0.005$; ★$P < 0.01$ and $^{\times}P < 0.05$).

**Table V** Pharmacokinetic parameters of CPT-11 and SN-38 determined on day 8

| Dose (mg m$^{-1}$) | No. of patients | CPT-11 Cmax[a] μg ml$^{-1}$ | CPT-11 AUC[b] μg ml$^{-1}$ × h | CPT-11 $t_{1/2}$[c] h | CPT-11 MRT[d] h | CPT-11 CLp[e] l m$^{-2}$ per h | CPT-11 Vd[f] l m$^{-2}$ | SN-38 Cmax ng ml$^{-1}$ | SN-38 AUC ng ml$^{-1}$ × h | SN-38 $t_{1/2}$ h |
|---|---|---|---|---|---|---|---|---|---|---|
| 80 | 7 | 0.95 ± 0.16[g] | 4.98 ± 1.24 | 12.26 ± 8.60 | 6.25 ± 1.25 | 17.21 ± 5.54 | 269.04 ± 157.70 | 13.23 ± 4.18 | 216.0 ± 97.3 | 25.01 ± 15.26 |
| 90 | 3 | 0.95 ± 0.12 | 5.94 ± 1.74 | 9.25 ± 2.40 | 6.96 ± 0.59 | 15.99 ± 4.40 | 221.37 ± 114.38 | 29.03 ± 10.88 | 340.1 ± 140.0 | 16.00 ± 3.22 |
| Total | 10 | – | – | 11.36 ± 7.26 | 6.46 ± 1.11 | 16.84 ± 5.01 | 254.74 ± 141.48 | – | – | 22.31 ± 13.28 |

[a]Peak plasma concentration. [b]Area under the plasma concentration-time curve. [c]Terminal half-life. [d]Mean residence time. [e]Total plasma clearance. [f]Total volume of distribution. [g]Mean ± s.d.

**Table VI** Treatment results for all patients by dose level

| *Dose level* | *No. of patients* | *No. of responders (%)* | *Time to remission days* | *Response duration days* |
|---|---|---|---|---|
| 1 | 3 | 2 (67) | 42,44 | 85,170 |
| 2 | 8 | 3 (38) | 41 (30–42)[a] | 158 (92–208) |
| 3 | 3 | 1 (33) | 30 | 98 |
| Total | 14 | 6 (43) | 42 (30–44) | 128 (85–208) |

[a]Median (range).

level. The median time required to reach remission was 42 days (range: 30 to 44 days). These were five partial responses and one complete response ranging from 85 to 208 days in duration (median: 128 days). Eight patients showed no change, and none of the patients showed disease progression. The response rates for NSCLC and SCLC were 33% (four of 12 patients) and 100% (two of two patients), respectively. Two (29%) partial responses and one complete response (14%) were observed in the seven patients with stage IIIB disease for an overall response rate of 43%. A partial response was obtained in three (43%) of the seven patients with stage IV disease.

### Discussion

Preclinical studies demonstrated that CPT-11 and its major active metabolite (SN-38) may be synergistic with cisplatin (Takada *et al.*, 1992). The starting dose of CPT-11 for the present study was chosen on the basis of the results of a previous phase I trial in patients with advanced NSCLC at our institution (Masuda *et al.*, 1992*b*). The recommended dose of CPT-11 in combination with 80 mg $m^{-2}$ of cisplatin was found to be 60 mg $m^{-2}$, with leukopenia and diarrhea being the dose-limiting toxicities. The dose intensity of CPT-11 achieved with this schedule was only 45% of that reported for a single-agent administration as a weekly intravenous infusion Negoro *et al.*, 1991*a*), showing that the combined administration of full doses of both these drugs was not feasible. In view of the high single-agent activity of CPT-11 against SCLC (Negoro *et al.*, 1991*b*; Masuda *et al.*, 1992*a*) and NSCLC (Fukuoka *et al.*, 1992), a regimen with a higher dose of this agent and a lower dose of cisplatin seemed likely to be more active.

The dose-limiting toxic effect of this combination was severe diarrhea and myelosuppression was not a dose-limiting problem in this trial (Table III). Because all three patients given 90 mg $m^{-2}$ of CPT-11 developed grade 3 diarrhea during their first course of therapy, we stopped further dose escalation of this agent. Therefore, a dose of 80 mg $m^{-2}$ of CPT-11 given intravenously on days 1, 8, and 15 plus 60 mg $m^{-2}$ of cisplatin every 4 weeks is the recommended starting dose for future Phase II studies in patients who have had no prior chemotherapy. Reduction of the cisplatin dose to 60 mg $m^{-2}$ (a 25% decrease in the cisplatin dose intensity) allowed in the safe administration of CPT-11 at 80 mg $m^{-2}$ without dose-limiting diarrhea or leukopenia and resulted in a 33.3% dose intensification of the latter agent compared with the previous Phase I trial.

Although marked interpatient variability of gastrointestinal toxicity was observed in this study, which is a well known feature of CPT-11 (Negoro *et al.*, 1991*a*; Negoro *et al.*, 1991*b*; Fukuoka *et al.*, 1992; Masuda *et al.*, 1992*a*), it seems likely that development of diarrhea was related to the pharmacokinetic behavior of SN-38 as reported by Sasaki *et al.* (Sasaki *et al.*, 1992), especially to the Cmax of SN-38 in this trial. The abrupt increase in the frequency of diarrhea observed on this trial with the modest increase in the CPT-11 dose from 80 mg $m^{-2}$ to 90 mg $m^{-2}$ may be explained by the marked increase of this metabolite. In our previous phase I study for single agent administration as a weekly infusion (Negoro *et al.*, 1991*a*), the AUC values of CPT-11 were 2.97, 6.37, 9.17, and 11.29 μg $ml^{-1}$ × hours after administration of 50, 100, 125, and 150 mg $m^{-2}$ of CPT-11, respectively, showing a nonlinear pharmacokinetics of CPT-11. The AUC values of SN-38 increased from 117 to 252 ng $ml^{-1}$ × hours with a dose intensification of CPT-11 from 50 to 125 mg $m^{-2}$. The AUC values of SN-38 tended to increase with the increase of the CPT-11 dose in that trial although there was wide variability in each patient. Therefore, the extraordinary increase in the plasma SN-38 levels with the slight increase in the CPT-11 dose in this trial was an unexpected event. These results strongly suggest the pharmacokinetic interaction between CPT-11 and cisplatin although the precise mechanism of this unexpected increase in SN-38 remains to be elucidated. In any case, if SN-38 plays an essential role in the onset of diarrhea, the activity of carboxylesterase (which catalyses the transformation of CPT-11 to SN-38) may be an important determinant of toxicity. Carboxylesterase shows different levels of expression in different species and different organs. In contrast with rats (Tsuji *et al.*, 1991), metabolism in human plasma is unlikely to contribute to the formation of SN-38, so metabolism in the liver and gastrointestinal tract epithelium may play a predominant role in the toxicity of CPT-11. If so, the prediction of which patients will experience severe toxicity is likely to be difficult, because we do not have any way to determine the enzyme activity in the liver or gastrointestinal tract. Sandostatin was reported to be effective for the diarrhea due to chemotherapy or radiotherapy (Kennedy *et al.*, 1990; Petrelli *et al.*, 1992), but the severe diarrhea observed in this trial did not respond to subcutaneous sandostatin in all of three patients treated. This may reflect a difference in the mechanism of diarrhea caused by a combination of CPT-11 and cisplatin and that seen as a complication of pelvic radiotherapy or of chemotherapy with 5-fluorouracil. Since sandostatin was ineffective in reducing diarrhea, the use of high-dose loperamide or more potent anti-diarrheal agents should be investigated to control severe diarrhea induced by this protocol. In addition, further studies to elucidate the precise mechanisms by which CPT-11 causes diarrhea are needed.

In summary, this study has shown that CPT-11 can be given at 80% of the recommended single-agent dose in combination with 60 mg $m^{-2}$ of cisplatin, while achieving acceptable toxicity. The major dose-limiting toxicity of this combination was diarrhea. In this Phase I study of 14 patients with advanced lung cancer, we observed five (36%) partial responses and one (7%) complete response, for an overall response rate of 43%. For future phase II studies, the recommended doses are 80 mg $m^{-2}$ of CPT-11 (days 1, 8, and 15) plus 60 mg $m^{-2}$ of cisplatin (day 1) at 4-week intervals. A Phase II trial of this regimen in previously untreated SCLC patients would be appropriate.

This work was supported in part by a Grant-in-Aid from the Japanese Ministry of Health and Welfare for the Comprehensive 10-year Strategy for Cancer Control, by a Grant-in-Aid for Cancer Research from the Japanese Ministry of Health and Welfare (2-S-1, 3-42), and by a grant from Daiichi Pharmaceutical Co., Ltd. (Tokyo, Japan).

We wish to thank Dr Hiroshi Komatsu, Mr Fumiyasu Fukuda and Mr Kyoji Tamanoi for their help with data collection and the pharmacokinetic analysis, and Drs Masaaki Kawahara and Masunari Yamamoto for their extramural review of this study.

### References

CHABNER, B.A. & MYERS, C.E. (1989). Clinical pharmacology of cancer chemotherapy. In *Cancer: Principles and Practice of Oncology*. DeVita, V.T., Hellmann, S. & Rosenberg, S.A. (ed, 4rd ed. pp. 349–395. Philadelphia: J.B. Lippincott Company.

FUKUOKA, M., NIITANI, H., SUZUKI, A., MOTOMIYA, M., HASEGAWA, K., NISHIWAKI, Y., KURIYAMA, T., ARIYOSHI, Y., NEGORO, S., MASUDA, N., NAKAJIMA, S. & TAGUCHI, T. (1992). Phase II study of CPT-11, a new derivative of camptothecin, for previously untreated non-small-cell lung cancer. *J. Clin. Oncol.*, **10**, 16–20.

HERTZBERG, R.P., CARANFA, M.J. & HECHT, S.M. (1989). On the mechanism of topoisomerase I inhibition by camptothecin: evidence for binding to an enzyme-DNA complex. *Biochemistry*, **28**, 4629–4638.

HSIANG, Y.H. & LIU, L.F. (1988). Identification of mammalian DNA topoisomerase I as an intracellular target of the anticancer drug camptothecin. *Cancer Res.*, **48**, 1722–1726.

HSIANG, Y.H., HERTZBERG, R., HECHT, S. & LIU, L.F. (1985). Camptothecin induces protein-linked DNA breaks via mammalian DNA topoisomerase I. *J. Biol. Chem.*, **260**, 14873–14878.

KANEDA, N., NAGATA, H., FURUTA, T. & YOKOKURA, T. (1990). Metabolism and pharmacokinetics of the camptothecin analogue CPT-11 in the mouse. *Cancer Res.*, **50**, 1715–1720.

KENNEDY, P., PRESANT, C.A., BLAYNEY, D., WISEMAN, C., KING, M. & GALA, K. (1990). Sandostatin (S) therapy for chemotherapy (CT) and radiotherapy (RT) related diarrhea (D). *Proc. Am. Soc. Clin. Oncol.*, **9**, 324 (abstract).

KUNIMOTO, T., NITTA, K., TANAKA, T., UEHARA, N., BABA, H., TAKEUCHI, M., YOKOKURA, T., SAWADA, S., MIYASAKA, T. & MUTAI, M. (1987). Antitumor activity of 7-ethyl-10-[4-(1-piperidino)-1-piperidino]carbonyloxy-camptothecin, a novel water-soluble derivative of camptothecin, against murine tumors. *Cancer Res.*, **47**, 5944–5947.

MASUDA, N., FUKUOKA, M., KUSUNOKI, Y., MATSUI, K., TAKIFUJI, N., KUDOH, S., NEGORO, S., NISHIOKA, M., NAKAGAWA, K. & TAKADA, M. (1992*a*). CPT-11: a new derivative of camptothecin for the treatment of refractory or relapsed small-cell lung cancer. *J. Clin. Oncol.*, **10**, 1225–1229.

MASUDA, N., FUKUOKA, M., TAKADA, M., KUSUNOKI, Y., NEGORO, S., MATSUI, K., KUDOH, S., TAKIFUJI, N., NAKAGAWA, K. & KISHIMOTO, S. (1992*b*). CPT-11 in combination with cisplatin for advanced non-small cell lung cancer. *J. Clin. Oncol.*, **10**, 1775–1780.

MATSUZAKI, T., YOKOKURA, T., MUTAI, M. & TSURUO, T. (1988). Inhibition of spontaneous and experimental metastasis by a new derivative of camptothecin, CPT-11, in mice. *Cancer Chemother. Pharmacol.*, **21**, 308–312.

MOUNTAIN, C.F. (1986). A new international staging system for lung cancer. *Chest*, **89**, 225S–233S.

NEGORO, S., FUKUOKA, M., MASUDA, N., TAKADA, M., KUSUNOKI, Y., MATSUI, K., TAKIFUJI, N., KUDOH, S., NIITANI, H. & TAGUCHI, T. (1991*a*). Phase I study of weekly intravenous infusions of CPT-11, a new derivative of camptothecin, in the treatment of advanced non-small-cell lung cancer. *J. Natl Cancer Inst.*, **83**, 1164–1168.

NEGORO, S., FUKUOKA, M., NIITANI, H. & TAGUCHI, T. (1991*b*). Phase II study of CPT-11, new camptothecin derivative, in small cell lung cancer (SCLC). *Proc. Am. Soc. Clin. Oncol.*, **10**, 241 (abstract).

OHNO, R., OKADA, K., MASAOKA, T., KURAMOTO, A., ARIMA, T., YOSHIDA, Y., ARIYOSHI, H., ICHIMARU, M., SAKAI, Y., OGURO, M., ITO, Y., MORISHIMA, Y., YOKOMAKU, S. & OTA, K. (1990). An early phase II study of CPT-11: a new derivative of camptothecin, for the treatment of leukemia and lymphoma. *J. Clin. Oncol.*, **8**, 1907–1912.

PETRELLI, N., RODRIGUEZ-BIGAS, M., CREAVEN, P. & RUSTUM, Y. (1992). Efficacy of somatostatin analogue (SMS), sandostatin, for treatment of chemotherapy induced diarrhea in colorectal cancer. *Proc. Am. Soc. Clin. Oncol.*, **11**, 170 (abstract).

SASAKI, Y., MORITA, M., MIYA, T., SHINKAI, T., EGUCHI, K., TAMURA, T., OHE, Y. & SAIJO, N. (1992). Pharmacokinetic (PK) and pharmacodynamic (PD) analysis of CPT-11 and its active metabolite SN-38. *Proc. Am. Soc. Clin. Oncol.*, **11**, 111 (abstract).

SHIMADA, Y., YOSHINO, M., WAKUI, A., NAKAO, I., FUTATSUKI, K., SAKATA, Y., KAMBE, M., TAGUCHI, T. & CPT-11 GASTROINTESTINAL CANCER STUDY GROUP (1991). Phase II study of CPT-11, new camptothecin derivative, in the patients with metastatic colorectal cancer. *Proc. Am. Soc. Clin. Oncol.*, **10**, 135 (abstract).

TAKADA, M., FUKUOKA, M., KUDOH, S., MASUDA, N., NAKAGAWA, K. & KISHIMOTO, S. (1992). Synergistic effects of CPT-11 and cisplatin or etoposide on human lung cancer cell lines and xenografts in nude mice. *Proc. Am. Assoc. Cancer Res.*, **33**, 226 (abstract).

TAKEUCHI, S., TAKAMIZAWA, H., TAKEDA, Y., OKAWA, T., TAMAYA, T., NODA, K., SUGAWA, T., SEKIBA, K., YAKUSHIJI, M., TAGUCHI, T. & CPT-11 STUDY GROUP ON GYNECOLOGIC MALIGNANCY (1991). Clinical study of CPT-11, camptothecin derivative, on gynecological malignancy. *Proc. Am. Soc. Clin. Oncol.*, **10**, 189 (abstract).

TSUJI, T., KANEDA, N., KADO, K., YOKOKURA, T., YOSHIMOTO, T. & TSURU, D. (1991). CPT-11 converting enzyme from rat serum: purification and some properties. *J. Parmacobio-Dyn.*, **14**, 341–349.

WORLD HEALTH ORGANIZATION (1979). WHO Handbook for Reporting Results of Cancer Treatment. WHO Offset Publication No. 48, Geneva, Switzerland, World Health Organization.